# *Lactobacillus* spp. attenuate antibiotic-induced immune and microbiota dysregulation in honey bees

**DOI:** 10.1038/s42003-020-01259-8

**Published:** 2020-09-25

**Authors:** Brendan A. Daisley, Andrew P. Pitek, John A. Chmiel, Shaeley Gibbons, Anna M. Chernyshova, Kait F. Al, Kyrillos M. Faragalla, Jeremy P. Burton, Graham J. Thompson, Gregor Reid

**Affiliations:** 1 Centre for Human Microbiome and Probiotic Research, Lawson Health Research Institute, London, ON Canada; 2 Department of Microbiology and Immunology, The University of Western Ontario, London, ON Canada; 3 Department of Biology, The University of Western Ontario, London, ON Canada; 4 Department of Surgery, The University of Western Ontario, London, ON Canada

**Keywords:** Bacterial host response, Antimicrobial responses, Microbiome, Bacterial pathogenesis, Microbial ecology

## Abstract

Widespread antibiotic usage in apiculture contributes substantially to the global dissemination of antimicrobial resistance and has the potential to negatively influence bacterial symbionts of honey bees (*Apis mellifera*). Here, we show that routine antibiotic administration with oxytetracycline selectively increased *tetB* (efflux pump resistance gene) abundance in the gut microbiota of adult workers while concurrently depleting several key symbionts known to regulate immune function and nutrient metabolism such as *Frischella perrera* and *Lactobacillus* Firm-5 strains. These microbial changes were functionally characterized by decreased capped brood counts (marker of hive nutritional status and productivity) and reduced antimicrobial capacity of adult hemolymph (indicator of immune competence). Importantly, combination therapy with three immunostimulatory *Lactobacillus* strains could mitigate antibiotic-associated microbiota dysbiosis and immune deficits in adult workers, as well as maximize the intended benefit of oxytetracycline by suppressing larval pathogen loads to near-undetectable levels. We conclude that microbial-based therapeutics may offer a simple but effective solution to reduce honey bee disease burden, environmental xenobiotic exposure, and spread of antimicrobial resistance.

## Abstract (web summary)

Daisley et al. show that antibiotic treatment with oxytetracycline impairs the gut microbiota and immune system of honey bees, and reduces capped brood counts. They also show that supplementation with lactobacilli during antibiotic recovery can reverse the harmful effects of the antibiotic treatment. Their findings offer a simple microbial-based solution that aims to reduce honey bee disease burden, environmental pollution by xenobiotics, and spread of antimicrobial resistance.

## Introduction

Pathogens are considered one of the largest contributing factors in the global decline of honey bees (*Apis mellifera*) and other wild pollinators, which together help support agricultural economies and international food supplies^1–3^. Managed honey bees represent a substantial fraction of total pollinators and are an important reservoir of enzootic pathogens that can affect disease epidemiology in various animal communities^4^. To address this issue, beekeepers frequently administer antibiotics to their hives in an attempt to prevent or reduce disease occurrence and control intraspecies pathogen transmission between nearby apiaries.

Oxytetracycline (OTC), tylosin, and fumagillin are the most commonly used antibiotics in apiculture^5^. Tetracycline-based agents are of particular concern given their extensive usage in human medicine and as bacteriostatic feed additives in livestock. Though indispensable under certain circumstances, overuse of tetracyclines pollutes the environment and inadvertently leads to the accumulation of antibiotic-resistance genes in many bacteria, including a multitude of pathogens relevant to human and honey bee health^6^. Antibiotic exposure can also negatively impact key symbiotic bacteria within the microbiota (community of microorganisms residing on or within a multicellular organism) of honey bees^7^. This compromises overall health status in hives as the distinct microbiota in healthy bees is crucial to host metabolic competency, immune regulation, growth and development, and resistance towards parasites and pathogens^8^.

In many parts of the world, prophylactic usage of OTC is recommended under best management practices for beekeepers in the prevention of foulbrood diseases caused by *Paenibacillus larvae* and *Melissococcus plutonius*^9^. In the case of *P. larvae*, which causes American foulbrood (AFB), there have been reports of widespread tetracycline resistance occurring since the early 2000’s^10^. Identical sequence homology of tetracycline-resistance loci found in *P. larvae* and core members of the honey bee microbiota suggests that there is horizontal gene transfer between commensals and pathogens via mobile genetic elements such as transposons and plasmids^11^. Importantly, despite susceptibility or resistance, OTC is unable to treat *P. larvae* spores in the comb, which can act as a source of reinfection. To address these issues and guard against the potentially rapid evolution of resistance, it is prudent to test alternative control measures, which so far include hygienic breeding^12^, use of bioactive essential oils^13^, bacteriophage therapy^14^, synthetic indoles with anti-germination properties^15^, and supplementation of hives with lactic acid bacteria^16–18^. The promise of this latter approach is up-held by studies demonstrating that lactobacilli can promote insect innate immunity and detoxification^19–21^, extend longevity of adult honey bees^22^, and stimulate queen brood production^23,24^.

In our previous work, we demonstrated that pollen patty hive supplements infused with three select strains of lactobacilli (*Lactobacillus rhamnosus* GR-1, *Lactobacillus plantarum* Lp39, and *Lactobacillus kunkeei* BR-1; LX3) could reduce *P. larvae* loads in honey bee hives experiencing active AFB outbreak and improve honey bee survival towards *P. larvae* infection in vitro^25^. Here, we evaluated OTC treatment efficacy in subclinically infected hives and characterized how routine exposure to this common antibiotic impacts immune and microbiota dynamics in honey bees. In addition, we evaluated how treatment augmentation with LX3 following OTC exposure influences antibiotic recovery rates as evaluated by immune functionality, microbial homeostasis, and hive productivity.

## Results

### LX3 enhances larval pathogen eradication by antibiotics

**Fig. 1:**
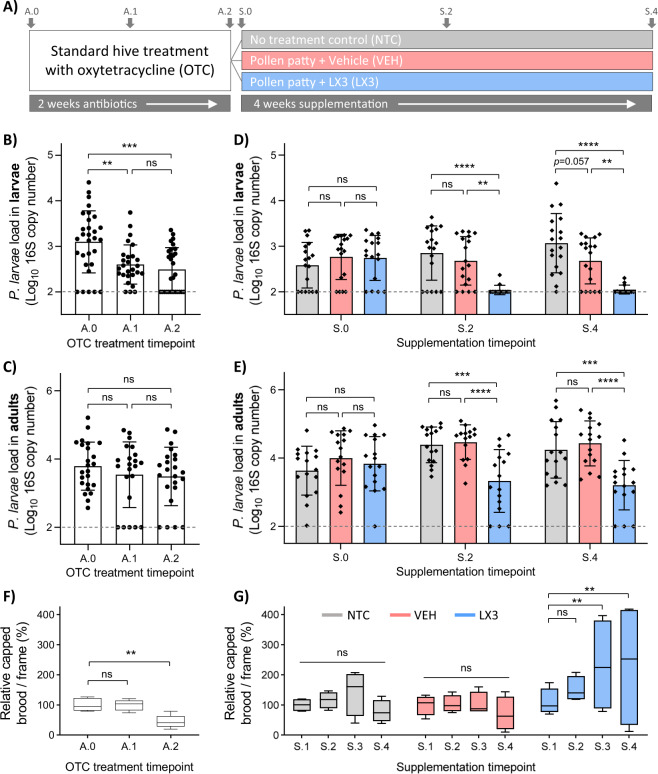
LX3 enhances larval pathogen eradication by antibiotics. Experimental hives were subjected to standard antibiotic treatment with oxytetracycline (OTC) for 2 weeks and then supplemented for 4 weeks with either pollen patties containing LX3 (LX3) or pollen patties containing vehicle (VEH). No treatment control (NTC) hives received no further treatment after OTC. **a** Schematic diagram outlining the experimental design. **b**, **c** Molecular-based quantification of *P. larvae* in honey bee larvae (whole body) and adults (dissected abdomen) collected just prior to the start of OTC exposure (A.0), and then after 1 (A.1) and 2 (A.2) weeks of exposure. Data are depicted as median ± 95% confidence intervals (Kruskal–Wallis with Dunn’s multiple comparisons) at different time points. Each data point represents either one individual (adults) or three pooled individuals (larvae) sampled equally from a total of *n* = 6 hives. **d**, **e** Molecular-based quantification of *P. larvae* in larvae (whole body) and adults (dissected whole abdomens) at the start of the supplementation period (S.0; corresponding to 3 days post A.2 time point), and then after 2 (S.2) and 4 (S.4) weeks. Data are depicted as mean ± standard deviation (two-way ANOVA with Sidak’s multiple comparisons) at different time points with each data point representing either one individual (adults) or three pooled individuals (larvae) sampled equally from *n* = 4 hives per treatment group. **f**, **g** Capped brood counts during OTC treatment (*n* = 6 hives) and subsequent supplementation period (*n* = 4 hives per treatment group). Data represents the median (line in box), IQR (box), and minimum/maximum (whiskers) of relative change in brood counts normalized by hive. Statistics shown for one-way and two-way ANOVA, respectively, with Sidak’s multiple comparisons for both. **P < 0.01, ***P < 0.001, ****P < 0.0001, ns not significant.

Prophylactic administration of OTC to honey bees is a common practice in beekeeping for the prevention of AFB. To evaluate the efficacy of this long-standing apiculture management strategy, we monitored a 2-week treatment regimen with OTC under natural field conditions in honey bee hives experiencing low-grade chronic infection with *P. larvae* (Fig. 1a). Using a qPCR-based approach to enumerate pathogen load, *P. larvae* abundance was found to be significantly lower in honey bee larvae (primary target of AFB) at week 1 and week 2 of OTC treatment (Kruskal–Wallis with Dunn’s multiple comparisons, *P* = 0.0071 and *P* = 0.0005, respectively) compared to baseline levels at day 0 (Fig. 1b). In contrast, no observable differences in *P. larvae* abundance were found in adult honey bees (active vector of AFB) at any time point during this treatment (Kruskal–Wallis with Dunn’s multiple comparisons, *P* = 0.9999, *P* = 0.6367, respectively; Fig. 1c).

A robust body of scientific evidence shows that supplementation with probiotic *Lactobacillus* spp. can augment the effects of certain antibiotics and attenuate antibiotic-induced dysbiosis in humans and other animals^26–29^. Testing this in honey bees, it was found that larval samples from LX3-treated hives exhibited significantly lower levels of *P. larvae* at week 2 and week 4 compared to both no treatment control (NTC; *P* < 0.0001 for both) and vehicle-treated (*P* = 0.0011 and *P* = 0.0014) hives, respectively (two-way analysis of variance [ANOVA] with Sidak’s multiple comparisons; Fig. 1d). The NTC group also demonstrated a trend towards higher *P. larvae* loads in larval samples compared to the vehicle group at week 4 (two-way ANOVA with Sidak’s multiple comparisons, *P* = 0.0568). Similar results were observed in adults with samples from LX3-supplemented hives demonstrating a significantly lower *P. larvae* load compared to both the NTC group (*P* = 0.0002 and *P* = 0.0003) and vehicle-treated group (*P* < 0.0001 for both) at 2 and 4 weeks, respectively (two-way ANOVA with Sidak’s multiple comparisons; Fig. 1e).

To compare the effects of OTC and LX3 treatments on overall hive health, the coverage of capped brood on hive frames (an established metric for assessing colony strength and reproduction^30^) was measured weekly during experimentation. No observable differences were found in capped brood counts following 1 week of OTC treatment, whereas a significant reduction was found after 2 weeks (one-way ANOVA with Sidak’s multiple comparisons, *P* < 0.9999 and *P* = 0.0041, respectively) compared to pretreatment baseline measurements (Fig. 1f). In contrast, capped brood counts were significantly higher in LX3-treated hives at week 3 and 4 of the supplementation period (two-way ANOVA with Sidak’s multiple comparisons, *P* = 0.0071 and *P* = 0.0055, respectively), while no differences were found in NTC and vehicle-treated hives at comparable time points (Fig. 1g).

### Antibiotics reduce key immune regulators in the adult gut microbiota

Given the broad-spectrum activity of tetracyclines, we evaluated how OTC exposure might influence the symbiotic bacterial communities associated with honey bees. Total bacterial loads, as determined by qPCR-based molecular quantification, were significantly reduced in adult bees following 1 week of OTC treatment (*P* < 0.0001) and in larvae (*P* = 0.0421) after 2 weeks of treatment (Kruskal–Wallis tests with Dunn’s multiple comparisons; Supplementary Fig. 2). The nurse-aged adult bees sampled at the experimental start point (pre-OTC exposure) and on the final day of OTC treatment (post-OTC exposure) were chosen for 16S rRNA gene sequencing-based microbiota analysis due to their close physical proximity with brood, passive carriage of *P. larvae*, and well-balanced representation of overall hive microbial diversity^31^.

**Fig. 2:**
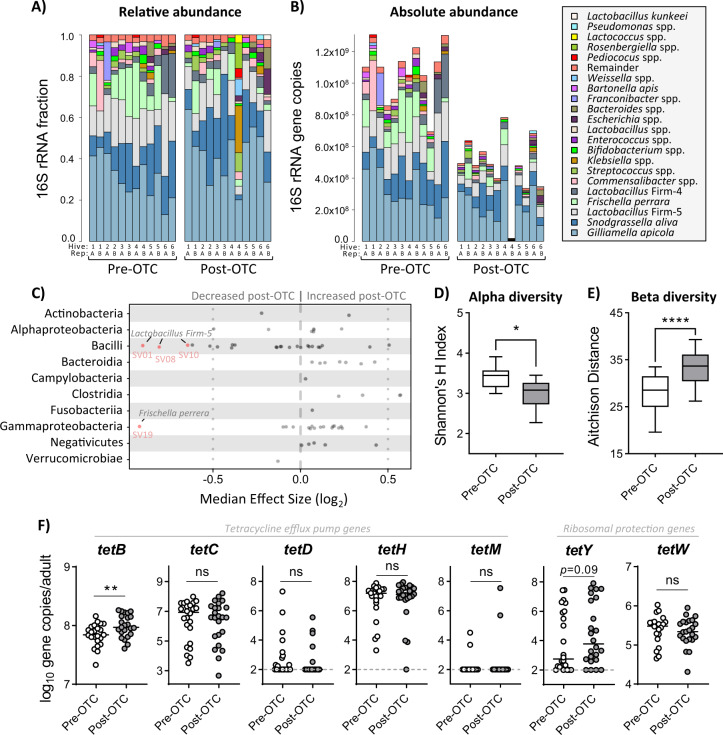
Antibiotics reduce key immune regulators in the adult gut microbiota. The gut microbiota of adult honey bees was analyzed before (Pre-OTC) and after (Post-OTC) hive administration with oxytetracycline. **a**, **b** Bar plots illustrating the relative and absolute abundance of bacterial species in the gut microbiota of adult honey bee samples as determined by V4 region 16S rRNA gene sequencing. Each bar represents a pooled sample of three adult guts collected from *n* = 6 hives, with two replicates performed for each hive. Absolute abundance of bacterial taxa was estimated by quantifying total 16S rRNA gene copy number via qPCR. **c** Strip chart showing differentially abundant taxa in the gut microbiota of OTC-exposed adult honey bees. Positive values indicate an increased relative abundance in response to OTC and negative values indicate a decreased relative abundance. Statistical inference was performed on centered log-ratio transformed read counts of sequence variants using ALDEx2 software in R. Features exceeding absolute effect size (>0.5) and *P* value (<0.05) thresholds are shown as red. **d**, **e** Alpha diversity (measured via Shannon’s H Index) and Beta diversity (measured via Aitchison’s distance between samples at different time points) of adult gut microbiota samples. Data represents the median (line in box), IQR (box), and minimum/maximum (whiskers) of respective microbiota diversity metrics with statistical comparisons shown for separate Wilcoxon tests. **f** Abundance of seven tetracycline-resistance loci in adult gut samples relative to the total number of 16S rRNA gene copies present. **P < 0.01 and ****P < 0.0001.

Bar plots shown in Fig. 2a, b visually represent the relative proportion and absolute abundance (adjusted according to qPCR-based quantification of total bacteria) of taxa in samples from pre- and post-OTC exposure, respectively. Adult gut samples collected post-OTC exposure revealed a significant reduction in a single amplicon sequence variant (SV) of *Frischella perrera* (SV19; Wilcoxon test with Benjamini–Hochberg [BH] multiple comparisons, *P* = 0.0043) and three unique SVs of *Lactobacillus* Firm-5 (SV01, SV08, and SV10; Wilcoxon tests with BH multiple comparisons, *P* = 0.0151, *P* = 0.0295, and *P* = 0.04217, respectively) compared to samples collected pre-OTC exposure (absolute effect >0.5; Fig. 2c). SV74 (*Lactobacillus* Firm-4), SV63 (*Bartonella apis*), and SV54 (*Lactobacillus* Firm-4) showed a trend towards decreased relative abundance following OTC exposure (absolute effect <0.5; Fig. 2c).

The compositional 16S rRNA gene sequencing dataset also demonstrated differences in alpha diversity (intraindividual) and beta diversity (interindividual) metrics of the adult gut microbiota following OTC exposure (Fig. 2d, e). Specifically, Shannon’s H index (balanced alpha diversity metric taking species abundance and evenness into account) was determined to be significantly lower following 2 weeks of OTC treatment (Wilcoxon test, *P* = 0.0273; Fig. 2d). These findings also corresponded with a decrease in microbiota stability as demonstrated by a significant increase in Aitchison distance (i.e. Euclidean distance after center log-ratio transforming SV read counts) between adults within the same treatment group (Wilcoxon test, *P* < 0.0001; Fig. 2e).

Next, to determine how the standard practice of treating hives with OTC may influence accumulation of antibiotic-resistance genes in the honey bee gut microbiota, we screened seven tetracycline-resistance loci that have been repeatedly detected in honey bee guts^6^. These loci included five tetracycline efflux pump genes (*tetB*, *tetC*, *tetD*, *tetH*, and *tetY*) and two ribosomal protection protein-encoding genes (*tetM* and *tetW*). The abundance of *tetB* was significantly higher (two-tailed *t* test*, P* = 0.0092) whereas *tetY* showed a trend towards increased abundance (two-tailed Mann–Whitney test, *P* = 0.0887) in post-OTC adult gut samples (two-tailed Mann–Whitney tests; Fig. 2f). No observable change in abundance were found for any of the remaining five tetracycline-resistance genes examined in this study (Fig. 2f). A positive association was identified between total Gammaproteobacteria (Pearson correlation, *r* = 0.888, *P* = 9.25 × 10^−17^) and *Gilliamella apicola* (Pearson correlation, *r* = 0.719, *P* = 1.23 × 10^−08^), but not *Frischella* *perrera* (Pearson correlation, *r* = −0.228, *P* = 0.124), and the presence of *tetB* (Supplementary Fig. 3).

### LX3 improves adult microbiota recovery post-OTC exposure

**Fig. 3:**
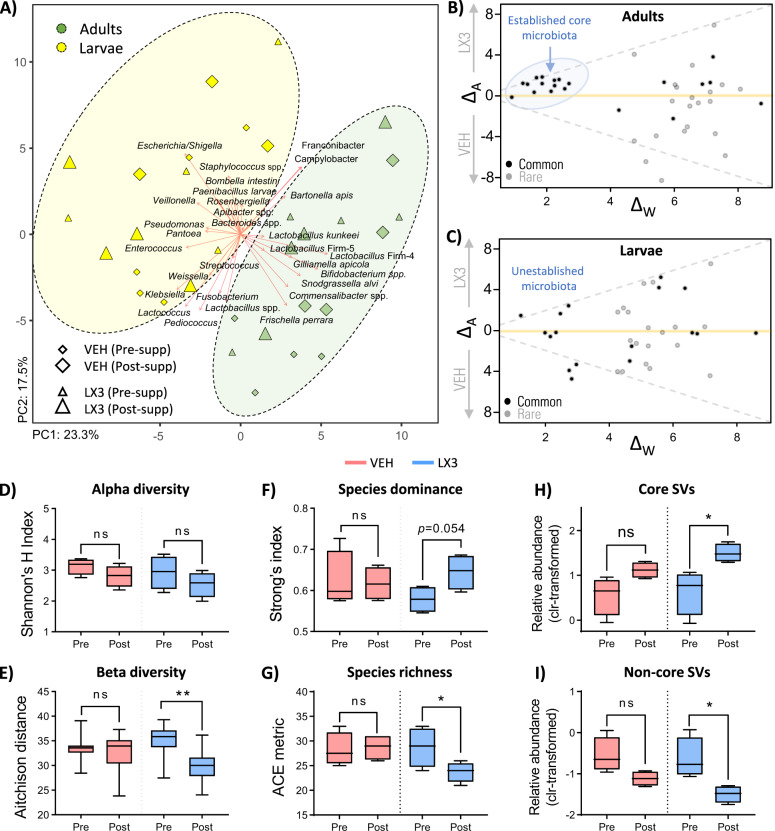
LX3 improves adult microbiota recovery post-OTC exposure. **a** Principle component analysis (PCA) plot of the honey bee microbiota from adult and larval samples before (Pre-supp) and after (Post-supp) the supplementation period. Sequence variants were collapsed at species-level identification, with clr-transformed Aitchison distances used as input values for PCA analysis. Distance between individual samples (points) represent the difference in microbiota composition between samples, with 40.8% of variance explained by the first two principle components shown. Strength of association for taxa are depicted by length of corresponding arrows. **b**, **c** ALDEx2 effect plots comparing differences in relative abundance of SVs between groups (ΔA) plotted against the variance, or within-group difference, in relative abundance for each SV (Δw). Low variance SVs that cluster tightly together in adult microbiota samples largely represent well-established core microbiota members (see Supplementary Data 1 for list of corresponding SVs). **d** Alpha diversity determined by Shannon’s H Index (accounting for abundance and evenness), **e** Beta diversity measured via Aitchison’s distance (representing within-group microbiota differences), **f** species dominance (or unevenness) measured via Strong’s Dw Index, and **g** species richness as determined using the abundance-based coverage estimator (ACE) metric in QIIME2. **h**, **i** Differential abundance analysis on adult gut samples between the relative abundance of all core cluster SVs grouped together compared to all noncore SVs grouped together. Data represents median (line in box), IQR(box), and minimum/maximum (whiskers) of clr-transformed relative abundances with statistical comparisons performed by Kruskal–Wallis test with Benjamini–Hochberg multiple comparisons. *P < 0.05, **P < 0.01, ns not significant.

In humans, probiotic therapy helps to encourage healthy remodeling of the microbiota and can improve recovery following antibiotics^32^. Here, we tested the ability of LX3 supplementation in honey bees to reduce *P. larvae* levels and restore microbiota homeostasis in adults and larvae following OTC exposure. As expected, principal component analysis showed clear separation between the microbiota composition of adult and larval samples (Fig. 3a). The largest influencers of separation that were positively associated with adult samples included core microbiota members such as *G. apicola*, *Snodgrassella alvi*, *F. perrara*, *Commensalibacter*, *Lactobacillus* Firm-4, and *Lactobacillus* Firm-5. In contrast, larval samples showed a positive association with mostly opportunistic bacteria including *Escherichia/Shigella*, *Staphylococcus*, *Enterococcus*, *Pseudomonas*, and *P. larvae* (Fig. 3a).

Using ALDEx2^33^ software, Bland–Altmann-like effect plots were generated to investigate the relationship between differences in relative abundance for each SV between treatment groups and the within-group variance of each SV. While no discernible differences were observed within larval samples with an underdeveloped microbiota composition, a tightly clustered group of 13 SVs with low variance were identified in adult samples (Fig. 3b, c). Notably, 12 out of 13 of these SVs trended towards increased abundance in the microbiota of LX3-supplemented adults (Fig. 3b) and consisted of several well-characterized honey bee core microbiota members including five SVs of *Lactobacillus* Firm-5, two SVs of *S. alvi*, two SVs of *G. apicola*, one SV of *Lactobacillus* Firm-4, one SV of *F.* *perrara*, and one SV of *Bifidobacterium* (see Supplementary Data 1 for full list of SVs). Corroborating these results, LX3 supplementation demonstrated a rescuing effect on microbiota stability as demonstrated by a significant decrease in compositional differences between adult microbiota samples (measured via Aitchison distance; *P* = 0.0012), whereas no changes were observed in vehicle-treated adults (Kruskal–Wallis test with BH multiple comparisons, *P* = 0.7570; Fig. 3e).

No differences in overall alpha diversity, as determined by Shannon’s H Index (accounting for species abundance and evenness), were detectable in either vehicle or LX3 treatment groups (Kruskal–Wallis test with BH multiple comparisons, *P* = 0.2985 and *P* = 0.2348, respectively; Fig. 3d). However, further investigation demonstrated that species richness alone (determined using the abundance-based coverage estimator [ACE] algorithm in QIIME2) was significantly lower (*P* = 0.0428) and that compositional dominance (or unevenness, as determined by Strong’s Dw Index) trended towards being higher (*P* = 0.0535) in LX3-treated adults compared to vehicle-treated adults (Kruskal–Wallis test with BH multiple comparisons; Fig. 3f, g). Since these data did not provide conclusive evidence as to whether core SVs (dominant microbiota members) or noncore SVs (rare species and transient opportunists) were responsible for the observed differences in diversity indices, a nested compositional analysis was performed on total relative abundance for each group. LX3-treated adults demonstrated a significant enrichment in core SVs (effect size = 1.3631) and a reduction in noncore SVs (effect size = −1.3860; Kruskal–Wallis test with BH multiple comparisons, *P* = 0.0225; Fig. 3h). In contrast, no change was seen in the total relative abundance of core SVs (effect size = 0.1579) or noncore SVs (effect size = −0.1654) in the vehicle-treated controls (Kruskal–Wallis test with BH multiple comparisons, *P* = 0.2046; Fig. 3i).

### LX3 strains are detectable in-hive members post-supplementation

**Fig. 4:**
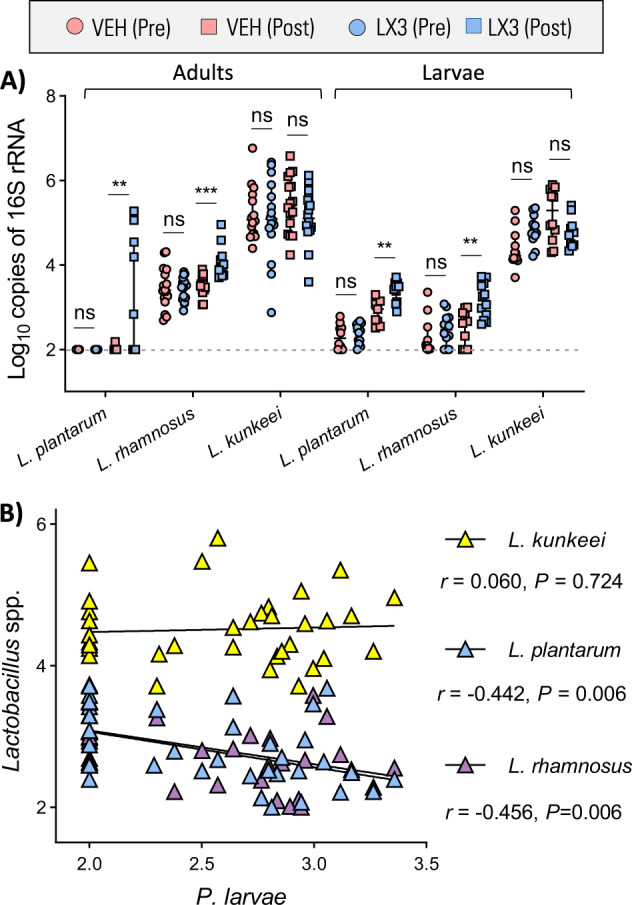
LX3 strains are detectable in-hive members post-supplementation. **a** Quantification of LX3 lactobacilli strains in honey bee adults and larvae before and after supplementation period. Data are depicted as mean ± standard deviation (two-tailed *t* tests) of bacterial abundances (determined via qPCR with species-specific primers) at different time points with each data point representing a single individual (*n* = 18 adult guts per treatment group at each time point) or a pooled sample of three larvae (*n* = 12 pooled samples for each treatment group at each time point). **b** Pearson correlation analysis between *P. larvae* and LX3 lactobacilli strains (quantified via qPCR) in honey bee larval samples. VEH = Pollen patty supplementation with vehicle, LX3 = Pollen patty supplementation with LX3. *P < 0.05, **P < 0.01 and ****P < 0.0001.

Since 16S rRNA gene sequencing is unable to resolve bacterial taxonomy at the species level, we performed qPCR-based quantification of Lp39, GR-1, and BR-1 using established primer sets^25^ to confirm that LX3 strains were being effectively dispersed throughout the hive as intended. LX3-supplemented adults and larvae were found to contain significantly higher levels of *L. plantarum* (multiple *t* tests, *P* = 0.0087 and *P* = 0.0035, respectively) and *L. rhamnosus* (multiple *t* tests, *P* = 0.0002 for both) compared to vehicle-treated control groups (Fig. 4a). In addition, evaluation of bacterial compositions in honey bee larval samples showed that abundance of *P. larvae* correlated inversely with *L. plantarum* (*r* = −0.442, *P* = 0.006) and *L. rhamnosus* (*r* = −0.456, *P* = 0.006), but not with *L. kunkeei* (*r* = 0.060, *P* = 0.724, Pearson correlations; Fig. 4b).

### LX3 upregulates both head and gut immunity in adult bees

**Fig. 5:**
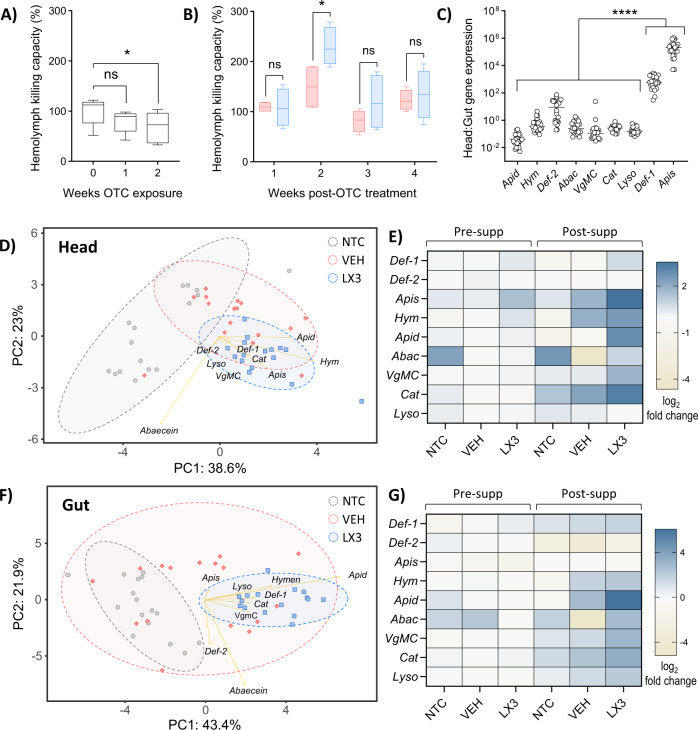
LX3 upregulates both head and gut immunity in adult bees. Adult hemolymph killing capacity against *A. globiformis* during **a** antibiotic treatment and **b** supplementation periods. Data represents the median (line in box), IQR (box), and minimum/maximum (whiskers) of hemolymph killing capacity for *n* = 6 hives (during antibiotic treatment) and *n* = 4 hives per treatment group (during supplementation period), respectively. Representative measurements for each hive at each time point were derived from a total of five randomly sampled adult nurse bees. Statistical analysis shown for one-way and two-way ANOVAs, respectively, with Sidak’s multiple comparisons. **c** Intraindividual head-to-gut gene expression ratios of nine innate immune- or antioxidant-related genes in adult honey bees. Gene expression was quantified by RT-qPCR with gut gene expression shown as relative to head gene expression. Data shown represents the mean ± standard deviation (one-way ANOVA with Sidak’s multiple comparisons) for *n* = 24 adults. PCA plots and heatmaps demonstrating innate immune- or antioxidant-related gene expression in **d**, **e** head and **f**, **g** gut samples of adult honey bees before (Pre-supp) and after (Post-supp) the supplementation period. Log2-transformed relative gene expression estimates (determined via qPCR) were used as input values for PCA analyses. Distance between individual samples (points) represent the difference in gene expression profiles for the nine immune or antioxidant genes shown, with 61.6% (heads) and 65.3% (guts) of variance explained by the first two principle components. Strengths of association for each gene are depicted by the length of corresponding arrows. Ellipses indicate 95% confidence intervals for each treatment group. NTC = no treatment control, VEH = pollen patty supplementation with vehicle, LX3 = pollen patty supplementation with LX3. *P < 0.05, ****P < 0.0001, ns not significant. Capped brood counts during OTC treatment (*n* = 6 hives) and subsequent supplementation period (*n* = 4 hives per treatment group). Data represents the median (line in box), IQR (box), and minimum/maximum (whiskers) of relative change in brood counts normalized by hive. Statistics shown for one-way and two-way ANOVA, respectively, with Sidak’s multiple comparisons for both. **P < 0.01, ***P < 0.001, ****P < 0.0001, ns not significant.

Repeated exposure to antibiotics can weaken immune defenses in honey bees—a phenomenon thought to be facilitated through the reduction of bacterial species important to immunoregulation^34^. Using an established zone-of-inhibition (ZOI) assay as a crude measure of immune function^35^, we assessed the inhibitory potential of honey bee-derived hemolymph against *Arthrobacter globiformis*. The antimicrobial capacity of adult hemolymph was found to be reduced by 31.27% (95% CI = 8.19–54.34%, one-way ANOVA with Sidak’s multiple comparisons, *P* = 0.0150) following 2 weeks of OTC treatment (Fig. 5a). In contrast, the antimicrobial capacity of hemolymph from LX3-supplemented adults was significantly increased by 121.30% (95% CI = 22.34–220.30%, two-way ANOVA with Sidak’s multiple comparisons, *P* = 0.0443) after 2 weeks in comparison to vehicle supplemented controls (Fig. 5b).

Previous work has shown that ex situ supplementation of LX3 to honey bee larvae can increase innate immune gene expression of several antimicrobial peptides (AMPs) that control susceptibility to *P. larvae* infection^25^. Here, we tested how LX3 supplemented directly to the hive impacted adult bee immunity. Expression of nine well-characterized innate immune- and antioxidant-related genes (*defensin-1*, *defensin-2*, *hymenoptaecin*, *apismin*, *apidaecin*, *VgMC*, *catalase*, and *lysozyme-1*) were measured in adult head and gut tissue as their respective anatomical sites are known to play a major role in social and individual immunity^36^. Exploratory analysis showed that basal gene expression levels in pre-supplemented adult bees demonstrated a vast enrichment of *defensin-1* (900.0 ± 163.6-fold higher) and *apismin* (297,292 ± 63,498-fold higher) in adults heads relative to gut expression, as compared to the other AMP genes examined (one-way ANOVA with Sidak’s multiple comparisons, *P* < 0.0001 for both; Fig. 5c)—suggesting that changes to the expression of these genes in the head, as opposed to the gut, might produce a more potent immune response and protective benefit at the colony level.

During the supplementation period, head expression of *defensin-1*, *apismin*, and *apidaecin* showed a significant increase over time in only the LX3-supplemented group (two-way ANOVAs with Sidak’s multiple comparisons, *P* = 0.0406, *P* < 0.0001, and *P* = 0.0004, respectively) whereas expression of *hymenoptaecin* increased in both LX3 and vehicle supplemented groups (two-way ANOVAs with Sidak’s multiple comparisons, *P* = 0.0240 and *P* = 0.0365, respectively; Supplementary Fig. 4A, C–E). All experimental groups showed an increase in head expression level of *catalase* (two-way ANOVA with Sidak’s multiple comparisons, *P* = 0.0050, *P* < 0.0001, and *P* < 0.0001, respectively) over time with no changes in expression of *defensin-2* or *lysozyme-1* (Supplementary Fig. 4B, H, I). Gut expression of *defensin-1*, *hymenoptaecin*, *apidaecin*, and *VgMC* were exclusively increased by LX3 supplementation (two-way ANOVAs with Sidak’s multiple comparisons, *P* = 0.0004, *P* = 0.0201, *P* < 0.0001, and *P* < 0.0001, respectively), whereas *lysozyme-1* and *catalase* expression increased in both vehicle (*P* = 0.0013 and *P* < 0.0001, respectively) and LX3 (*P* < 0.0001 for both) treatment groups (two-way ANOVA with Sidak’s multiple comparisons; Supplementary Fig. 4J, M, N, P–R and Supplementary Data 2). Relative expression of immune- and antioxidant genes in head and gut samples are visually summarized by the PCA plots and heatmaps in Fig. 5d–g.

### Correlations between host gene expression and bacterial loads

**Fig. 6:**
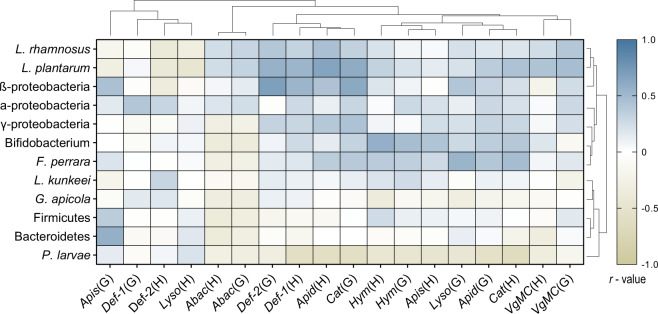
Correlations between host gene expression and bacterial loads. At the end of the 6-week experimental period, bacterial abundances in the guts of adult honey bees were compared with head and gut expression of nine immune- or antioxidant-related genes. Scale shown represents Pearson correlation coefficient, *r*, for *n* = 20–24 individual adults for each comparison. (G) = Gut gene expression, (H) = Head gene expression. The horizontal dendrogram acts to group host genes that covary in their expression patterns while the vertical dendrogram groups bacteria based on their co-occurring abundance relative to host gene expression. Both dendrograms were calculated using Euclidean distance and the complete ‘hclust’ function in R.

It has been known for decades now that probiotic bacteria can induce an immune response in honey bees^37^. In addition, recent evidence shows that host bacterial communities are selectively regulated by the innate immune system of honey bees, and that core microbiota members demonstrate a higher level of resistance to host AMPs than opportunistic bacterial pathogens^38^. Here, we examine the simultaneous relationship between bacterial abundances and immune- and antioxidant-related gene expression by using a dual extraction-based method to derive RNA and DNA from adult heads and guts. Experimental end point measurements at week 4 of the supplemental period demonstrated a negative Pearson correlation between total abundance of *P. larvae* and expression of *apidaecin* (*r* = −0.589, *P* = 0.004), *apismin* (*r* = −0.483, *P* = 0.023), *hymenoptaecin* (*r* = −0.460, *P* = 0.036), *defensin-1* (*r* = −0.599, *P* = 0.004), and *catalase* (*r* = −0.650, *P* = 0.001) in head samples, and with *apidaecin* (*r* = −0.559, *P* = 0.007), *hymenoptaecin* (*r* = −0.467, *P* = 0.029), and *catalase* (*r* = −0.589, *P* = 0.004) in gut samples (Fig. 6).

Assessment between the supplemented strains of lactobacilli and immune or antioxidant gene expression showed a positive relationship (Pearson correlations) between *L. plantarum* abundance and head expression of *apidaecin* (*r* = 0.653, *P* = 0.001), *defensin-1* (*r* = 0.526, *P* = 0.014), and *catalase* (*r* = 0.435, *P* = 0.043), as well as gut expression of *defensin-2* (*r* = 0.401, *P* = 0.008) *catalase* (*r* = 0.591, *P* = 0.004), and *VgMC* (*r* = 0.468, *P* = 0.028; Fig. 6). For *L. rhamnosus*, bacterial abundance was associated with increased head expression of *apidaecin* (*r* = *0.454*, *P* = 0.039) and a trend towards increased gut expression of *defensin-2* (*r* = 0.401, *P* = 0.072). No correlations were observed between gene expression and abundance of *L. kunkeei*. Core microbiota members demonstrated varying relationships with head and gut expression of immune- and antioxidant-related genes in adult honey bees. Total abundance of Alphaproteobacteria, Betaproteobacteria, Gammaproteobacteria, *Bifidobacterium*, and *F. perrara* clustered together (based on Euclidean distance of Pearson correlation coefficients) and were found to be mostly associated with increased AMP gene expression (Fig. 6). Oppositely, total abundance of Bacteroidetes, Firmicutes, and *G. apicola* clustered together and were mostly associated with an overall decrease in immune- and antioxidant-related gene expression irrespective of body site. A notable exception to these trends was the gut expression of *apismin* and *defensin-1* as well as the head expression of *defensin-2* and *lysozyme-1*, which clustered together (Euclidean distance matrix) based on similarities in gene expression patterns relative to bacterial abundances (Fig. 6). Pearson correlation coefficients (and associated statistics) for all relationships between bacterial abundances and host gene expression are provided in Supplementary Data 3.

## Discussion

This study demonstrated that in-hive supplementation with LX3 can augment *P. larvae* clearance rates during chronic low-grade infection and improve honey bee immunity following standard antibiotic treatment. Notably, the combination of OTC treatment followed by LX3 supplementation suppressed *P. larvae* more effectively than OTC treatment alone (to nearly undetectable levels) in honey bee brood. This combined treatment approach, but not antibiotics alone, also demonstrated the capacity to lower *P. larvae* loads in the gut microbiota of adult honey bees. Furthermore, LX3 treatment helped to partially restore antibiotic-induced deficits in-hive productivity, beta diversity of the gut microbiota in adult nurse bees, and immune responsiveness of adult-derived hemolymph. These findings expand on previous work in which Lp39, GR-1, and BR-1 strains of lactobacilli were shown to improve survival of laboratory-reared honey bee larvae in an acute infection model of *P. larvae*, as well as mitigate disease severity during an active AFB outbreak^25^.

Tetracycline-derived antibiotics have been shown to reduce the abundance^39^ and genetic diversity^7^ of core bacterial species in the gut microbiota of caged honey bee workers. Consistent with these reports, OTC directly administered to the hive altered the gut microbiota diversity of adult bees with significant decreases in relative abundances of *F. perrara* and *Lactobacillus* Firm-5 post-treatment (Fig. 2). Interestingly, metagenomic sequencing has demonstrated the genomes of Gammaproteobacteria (e.g. *F. perrara*) and Firmicutes (e.g. *Lactobacillus* Firm-5) to be specifically enriched with carbohydrate metabolism enzymes that can degrade mannose^40^—a prevalent plant-derived sugar known to be toxic to honey bees. In addition to the *direct* toxicity that antibiotics exert on honey bees^41^, our results suggest that antibiotic exposure may also *indirectly* increase mortality via depletion of symbionts crucial for the breakdown of toxic food components. The evaluation of LX3 supplementation in hives following cessation of OTC treatment elucidated a partial restorative effect on the adult gut microbiota (Fig. 3b). This effect was structurally characterized by a relative enrichment of core microbiota members and a reduction of transient opportunists (Fig. 3h, i). Together, these findings validate the negative and long-lasting implications of OTC exposure in honey bees while also highlighting a potential role for probiotics in safeguarding against the blooming effect of low-level pathobionts and transient opportunists following antibiotic exposure^42^.

Although widespread tetracycline resistance has been identified in honey bees^6^, there is lacking knowledge on how the adult gut metagenome may facilitate pathogen persistence during in-hive antibiotic treatment. This study demonstrated that OTC could effectively lower *P. larvae* loads in honey bee brood (unstable microbiota profile characterized by low abundance and diversity^43^), but not adult nurses (well-characterized microbiota profile with high abundance and diversity^44^) after 2 weeks of in-hive treatment (Fig. 1b, c). Similarly to the reduced diversity of tetracycline-resistance genes observed in OTC-exposed wax moths (*Galleria mellonella*)^45^, the relative abundance of five out of seven resistance genes (*tetC*, *tetD*, *tetH*, *tetM*, and *tetW*) in adult nurse bees remained unchanged or was diminished by OTC, while the relative abundance of *tetB* (and to a lesser extent, *tetY*) was increased (Fig. 2f). These findings suggest that the adult honey bee gut microbiota may provide *P. larvae* safe harborage against antibiotic exposure—potentially through polymicrobial synergy of multiple distinct resistance factors^46^ or via physical exclusion^47^ within the thick stratified biofilm known to be produced by *S. alvi* (existing in the bottom layer directly associated with host epithelium) and *G. apicola* (predominantly in the top layer) in the hindgut of bees^48^. Supporting this postulation, relative abundance of *G. apicola* was unaffected by OTC treatment (Fig. 2a–c) yet showed a strong positive association with *tetB*—an efflux pump-encoding gene which itself was shown to increase during OTC exposure (Fig. 2f). Presence of the *tetB* gene was confirmed in select *G. apicola* strains wkB1 and PEB0162^6^, though only ~18% of wild isolates are expected to possess tetracycline-resistance genes. Considering that *G. apicola* strains vary in their ability to beneficially regulate honey bee nutrition^49,50^, metabolism^40,51^, and microbiota composition^52,53^, the consequences of antibiotic selective pressures on intraindividual strain diversity merits further study.

An auxiliary repercussion of antibiotic-induced dysbiosis is perturbation of innate immunity. Adult bees exhibited a reduction in hemolymph antimicrobial capacity over time during the OTC treatment period (Fig. 5a), suggesting a disturbance in their microbial regulation. These findings support past observations of antibiotic-induced susceptibility to opportunistic infection by *Serratia marcescens*^39^—a Gram-negative bacterial pathobiont frequently associated with honey bees. Our current findings show that LX3 supplementation broadly upregulated expression of several AMPs (crucial immune effectors involved in pathogen defense) in the heads and guts of adult honey bees (Fig. 5d–g). *Defensin-1* is critical in controlling Gram-positive bacteria^54^ (including *P. larvae*^55^) whereas *apidaecin* and *hymenoptaecin* are most active against Gram-negative bacteria (including many opportunistic pathogens)^56,57^. Notably, expression of *apidaecin* increased by over fivefold in heads (primarily role in social immunity) and 50-fold in guts (primary role in individual immunity) after 4 weeks of LX3 supplementation, while no discernible changes were observed in vehicle and NTC groups.

In a recent report, Kwong et al. showed expression of *apidaecin* to be uniquely elevated in healthy adults in comparison to microbiota-depleted counterparts^38^. Moreover, three core phylotype strains of *G. apicola*, *S. alvi*, and *Lactobacillus* Firm-5 were found to upregulate *apidaecin* (and to a lesser extent *hymenoptaecin*) expression in gut tissue yet were largely resistant to both of these purified AMPs in vitro^38^. This suggests that host AMPs may play a role in shaping the microbiota through pathogen exclusion and selective refinement of evolutionarily adapted bacterial species. Supporting this theory, we found that intraindividual expression of *apidaecin* and several other key immune genes covaried in their expression patterns based on tissue type as well as the abundance of LX3 strains and core phylotype members in the gut (Fig. 6). Whereas for *P. larvae*, abundance in the adult gut was negatively associated with the expression of most AMPs tested, which suggests a robust host immune response is critical to suppressing this pathogen in the hive. The findings also suggest that the increase in core SVs and decrease in noncore SVs observed in LX3-treated adults (Fig. 3b) were likely a result of upregulated host AMPs (Fig. 5d–g). This evidence cumulatively supports that timely use of probiotics may help in the re-establishment of a healthy microbiota following OTC exposure and concurs with the proposition that immunostimulatory *Lactobacillus* spp. can ‘reset’ dysbiotic microbiota phenotypes^58^. Thus, while it has been long known that probiotics can induce an immune response in honey bees^37^, the functionality of probiotics in modulating AMPs with microbiota-shaping properties is only now becoming apparent. It is important to note, however, that LX3 supplementation was unable to fully restore microbiota composition to pre-OTC baseline levels in terms of overall diversity and structure during the time frame of this study. Though, a limitation to our findings is that low-level residues of antibiotics can persist in the hive for up to 3 months post-treatment^39^, which may have functionally impeded the corrective potential of treatment. Nevertheless, it remains plausible that targeted modulation of immune components may facilitate host-guided microbiota restoration following exposure to noxious chemicals distributed across apicultural and agricultural landscapes.

Resolving the mechanisms by which supplemental lactobacilli modulate honey bee immunity under normal field conditions, and the subsequent impact this has on their microbiota, is challenging due to the highly complex social interaction networks of adult workers and convoluted microbial dynamics in the hive. Though, innate immunity of insects is highly conserved^59^ with peptidoglycan recognition proteins (PGRPs) thought to play an important role in bacterial sensing via cell wall component recognition. Interestingly, some but not all *Lactobacillus* spp. produce diaminopimelic acid (DAP)-containing peptidoglycan^60^ which is known to activate Imd pathway signaling (leading to downstream production of AMPs) in insects via select PGRP binding^61^. Corroborating this, past work has shown that Lp39 (one of the LX3 strains used in this study) produces DAP-type peptidoglycan that can potently upregulate host AMP gene expression via Imd pathway signaling in *D. melanogaster*^19^. Alternatively, LGR-1 can modulate other divisions of insect innate immunity (e.g. Duox pathway signaling) and ameliorate neonicotinoid-induced suppression of host-generated microbicidal reactive oxygen species^21^. Its noteworthy to mention that honey bee-derived core lactobacilli members including *L. kunkeei*, *L. apinorum*, *L. mellifer*, *L. mellis*, *L. melliventris*, *L. kimbladii*, *L. helsingborgensis*, and *L. kullabergensis* all lack the ability to produce DAP-type peptidoglycan^60,62^. Accordingly, the absence of this crucial cell wall immune modulator may potentially explain the multiple recent failures at using honey bee-specific lactobacilli to reduce AFB disease severity^63,64^. Whether *L. kunkeei* BR-1 affected the overall immune-boosting performance of LX3 is undetermined, though it would be an interesting avenue for future studies given the opposing immune response to various lactobacilli-derived peptidoglycans observed in mouse models^65^.

In summary, this study has: (i) recapitulated the deleterious effects of antibiotics on the gut microbiota, immunity, and productivity of honey bees, (ii) substantiated the anti-*P. larvae* properties of LX3 supplemented to subclinically infected hives, and (iii) characterized the transcriptional response of several important immune- and antioxidant-related genes in response to hive supplementation with LX3. These results are not intended to refute the value of antibiotics or discourage their usage under appropriate circumstances. Instead, we suggest that exploiting the multipronged and evolutionarily adapted immune repertoire of honey bees via microbial-based therapeutics could augment current management strategies and offer a more effective method of controlling hive pathogens.

## Methods

### Culture conditions of bacterial strains

The three strains of lactobacilli used in this study were *L. plantarum* Lp39 (American Type Culture Collect [ATCC] 14917), *L. rhamnosus* GR-1 (ATCC 55826), and *L. kunkeei* BR-1 (honey bee-derived isolate from a healthy hive)^25^. Unless otherwise stated, routine culturing of these strains was performed under anaerobic conditions at 37 °C using de Man, Rogosa, and Sharpe (catalog number: 288130, BD Difco) broth or agar supplemented with 10 g/L D-fructose (catalog number: F-3510, Sigma-Aldrich; MRS-F). *A. globiformis* (ATCC 8010) utilized in hemolymph ZOI assays was routinely cultured aerobically at 37 °C using Luria-Bertani (catalog number: DF0446173, BD Difco; LB) broth or agar.

### Apiary conditions and experimental design

Two separate experiments were performed in two distinct apiaries maintained within a single geographic region near Western University (London, ON, Canada). These experimental apiaries were selected based on their geographic inclusion (<1 km away; a distance shown to be at risk of contracting high spore loads^66^) within a boundary assessed to have a recent increase of AFB incidence denoted through provincial apiary inspection by the Ontario Ministry of Agriculture and Food and Ministry of Rural Affairs (OMAFRA). Apiary A (*N* = 8 Carniolan hives) and apiary B (*N* = 8 Buckfast hives) were subjected to similar experimental designs between early summer (June–July) and late summer (August–September), respectively (Supplementary Fig. 1). In each case, all hives received standard dusting treatment with OTC hydrochloride (catalog number: 0223111, MEDIVET Pharmaceuticals) for 2 weeks according to manufacturer instructions. Two hives in each apiary were then left to freely interact with experimental hives in an effort to emulate realistic buffering conditions of undisturbed local neighboring colonies. The remaining six hives in each apiary were longitudinally monitored for an additional 4 weeks after being randomly assorted into the following experimental groups: (i) a NTC group that received no supplementation following administration of OTC, (ii) a vehicle control group (VEH) that received a standard 250 g pollen substitute patty (28.5 g of soy flour, 74.1 g of granulated sucrose, 15.4 g of debittered brewer’s yeast, 132.1 g of a 2:1 [w/v] simple sucrose-based syrup solution) with the addition of 4 mL phosphate-buffered saline (0.01 M) once per week, or (iii) a probiotic supplementation group (LX3) that received a 250 g pollen substitute patty infused with Lp39, GR-1, and BR-1 strains (each at a final concentration of 1 × 10^9^ colony forming units [CFU]/g) once per week. All larval samples in this study represent third-to-fifth instar, whereas adult samples represent nurse-aged workers found in close association with the brood. Sampling was performed in a manner to minimize the number of bees removed from the hive during experimentation. Hive tools were flame sterilized prior to use between each of the hives and sterile latex gloves were employed to prevent cross-contamination of LX3 strains and potential pathogens.

### Hive sampling procedures

For molecular quantification of *P. larvae* during OTC exposure, adults and larvae were sampled from *n* = 6 hives in apiary A with several replicates per hive. For adults, four samples from each hive were collected per time point (0, 1, and 2 weeks) with each replicate consisting of an individual dissected abdomen (*n* = 24 samples for each time point collected equally across the hives). For larvae, five samples from each hive were collected per time point (day 0, day 7, day 14) with each replicate consisting of a pooled sample of three whole larvae (*n* = 30 samples for each time point collected equally across the hives). Similarly, for quantification of *P. larvae* during the post-OTC supplementation period, adults and larvae were sampled from a total of *n* = 12 hives (six hives from apiary A [*n* = 2 NTC, *n* = 2 for vehicle treatment, *n* = 2 for probiotic treatment] and six hives from apiary B [*n* = 2 NTC, *n* = 2 for vehicle treatment, *n* = 2 for probiotic treatment]). For both adults and larvae, four samples from each hive were collected per time point (0, 2, and 4 weeks) with each replicate consisting of either an individual dissected abdomen or a pooled sample of three whole larvae, respectively (*n* = 48 adult samples and *n* = 48 larval samples collected equally across 12 hives at each time point).

For experiments evaluating the adult microbiota in response to OTC exposure, *n* = 6 hives from apiary A were sampled in biological duplicate (i.e. two samples from each hive with each replicate consisting of three nurse-aged adult guts pooled together) at two separate time points (pre-OTC and post-OTC), which represented a total of 72 individual bees sampled randomly and equally across the hives. The same sampling methodology was also implemented in experiments evaluating the adult and larval gut microbiota during the post-OTC supplementation period, for which a total of *n* = 8 hives were used (four hives from apiary A [*n* = 2 for vehicle treatment, *n* = 2 for probiotic treatment] and four hives from apiary B [*n* = 2 for vehicle treatment, *n* = 2 for probiotic treatment]).

For experiments characterizing the relationship between intraindividual bacterial abundances and host innate immune or antioxidant gene expression, adults were sampled at the final time point (week 4 of the supplementation period) from a total of *n* = 12 hives (six hives from apiary A [*n* = 2 NTC, *n* = 2 for vehicle treatment, *n* = 2 for probiotic treatment] and six hives from apiary B [*n* = 2 NTC, *n* = 2 for vehicle treatment, *n* = 2 for probiotic treatment]). Each hive was sampled in duplicate (i.e. two samples from each hive with each replicate consisting of a single individual nurse-aged adult bee) with each adult sample then subcategorized into head and gut groupings (*n* = 20–24 total paired head and gut samples used as not all variables measured were detectable in all samples).

### Molecular-based quantification of *P. larvae*

Larval (whole body) samples were evaluated as they represent the target developmental stage for *P. larvae* infection, whereas adults (dissected abdomen) are passive carriers of *P. larvae* and provide a good estimate of overall microbial diversity^31^. Respective samples were surface sterilized using 0.25% sodium hypochlorite and then rinsed in ddH_2_O for 30 s. DNA was then extracted from samples using the previously described CTAB method^53^. Bacterial loads were determined by qPCR using the Power SYBR Green PCR Master Mix kit (Applied Biosystems) following manufacturer’s instructions. Universal 16S rRNA gene and species-specific primer sets used in this study are listed in Supplementary Table 1. All qPCR reactions were performed in DNase- and RNase-free 384-well microplates on a QuantStudio 5 Real-Time PCR System (Applied Biosystems) and analyzed with associated QuantStudio Design and Analysis software. Copy numbers of target 16S rRNA genes were calculated as previously described using established primer efficiencies and limits of detection^25^.

### Enumeration of capped brood

To evaluate the efficacy of OTC and LX3 supplementation on hive health dynamics, the coverage of capped brood on hive frames (an established metric for assessing colony strength and reproduction^30^) was measured weekly during experimentation. Briefly, both sides of every frame in all experimental hives were photographed at the specified sampling time points and then semiautomatically counted using image analysis software as previously described^24^.

### Determination of in vitro antimicrobial capacity of adult hemolymph

Antimicrobial activity of adult hemolymph was determined using an established ZOI assay^35^ Prior to experimentation, the bacterial indicator stock of *A. globiformis* was grown aerobically in LB broth at 30 °C for 48 h to a final concentration of 1.6 × 10^9^ CFU/mL. Subsequently, 1 mL of broth culture was used to create a lawn of *A. globiformis* on LB agar plates. Next, flash frozen adult samples were thawed to 4 °C and hemolymph was extracted via centrifugation (1200 × *g* for 1 min) following the aseptic removal of antennae. Further, antimicrobial capacity was determined by plating 0.75 μL of hemolymph in preconstructed wells on agar plates that had been freshly seeded with a lawn of *A. globiformis*. The bottom of each plate was marked with a grid consisting of 12 squares, each 2 × 2 cm. Streptomycin (0.1 g/mL in 80% glycerol) was used at 1:200 and 1:300 dilutions as a positive control to account for batch variation among agar plates^35^. The agar plates were then incubated aerobically at 30 °C for 48 h prior to measurement of ZOI diameters for hemolymph or antibiotic control wells. ZOI was normalized by hive, and data are shown relative to experimental start points for OTC and LX3 treatment periods.

### Preparation of the 16S rRNA gene library

Targeted amplification of the V4 region of the bacteria 16S rRNA gene was achieved using established GOLAY-barcoded primers (5′–3′) ACACTCTTTCCCTACACGACGCTCTTCCGATCTNNNNxxxxxxxxxxxxGTGCCAGCMGCCGCGGTAA and (5′–3′) CGGTCTCGGCATTCCTGCTGAACCGCTCTTCCGATCTNNNNxxxxxxxxxxxxGGACTACHVGGGTWTCTAAT wherein ‘xxxxxxxxxxxx’ represents the sample-specific 12-mer barcode following the Illumina adapter sequence used for downstream library construction^67^. A BioMek Automated Workstation (Beckman Coulter) was then used to transfer 2 µL of sample DNA (5 ng/µL) to a 96-well 0.2-mL PCR plate containing 10 µL of each primer per well (3.2 pmol/µL). Next, 20 µL of GoTaq 2X Colorless Master Mix (Promega) was added to each well and plates were sealed using PCR-grade adhesive aluminum foil. PCR was then performed using a Prime Thermal Cycler (Technie) with the following reaction conditions: an initial activation step at 95 °C, followed by 25 cycles of 95 °C for 1 min, 52 °C for 1 min, and 72 °C for 1 min. After completion, the thermocycler was held at 4 °C, and amplicons were stored at −20 °C until further processing.

### Sequencing and analysis of the 16S rRNA gene dataset

Processing of amplicon libraries was conducted at the London Regional Genomics Centre (Robarts Research Institute, London, ON, Canada) in which amplicons were quantified using PicoGreen (Quant-It; Life Technologies, Burlington, ON, Canada), pooled at equimolar ratios, and sequenced on the MiSeq paired-end Illumina platform adapted for 2 × 250 bp paired-end chemistry. Sequence reads were then processed, aligned, and categorized using the DADA2 (v1.8) pipeline to infer exact amplicon SVs from the data^68^. Briefly, sequence reads were filtered (reads truncated after a quality score of ≤2 and forward/reverse reads truncated after 155/110 bases, respectively) and trimmed (10 bases off 5′ end of reverse reads) using optimized parameter settings as recommended. Next, sequence reads were de-replicated, de-noised, and merged using DADA2 default parameters. After omitting PCR blank control samples, the dataset used to assess the effects of OTC on the adult microbiota consisted of 24 nurse bee-derived gut samples and a total of 1,109,022 reads with an average count of 46,209 reads per sample. Following quality assurance measures described in the DADA2 pipeline^69^, an average of 13.94% reads were removed from each sample, leaving a total of 955,475 filtered reads. The dataset which evaluated the impact of probiotic treatment following antibiotic exposure consisted of 16 nurse bee-derived gut samples (372,198 total reads with an average of 23,262 reads per sample) and 16 larval samples (59,335 total reads with an average of 3708 reads per sample). Following quality control, an average of 11.63% and 31.29% reads were removed from adult and larval datasets, respectively. SV read counts were left in their unadulterated state for analysis (i.e. copy number was not corrected) to prevent additional noise in the dataset^70^. Taxonomic designations were assigned to SVs using the SILVA nonredundant training set (V132) configured for DADA2. Phylotype identity was determined by cross referencing with a previously established honey bee-specific seed alignment of 276 unique representatives^71^. Raw sequence reads are uploaded to the NCBI Sequence Read Archive and accessible under BioProject ID PRJNA610196.

### Simultaneous extraction of RNA and DNA from individual honey bees

Dissected head and abdomen samples from adult honey bees were surface sterilized using 0.25% sodium hypochlorite and then rinsed in ddH_2_O for 30 s. Subsequently, samples were homogenized in TRIzol (Invitrogen) by beat beating and RNA extracted following manufacturer’s instructions. Using the same samples, DNA was also extracted from the TRIzol homogenates using a back-extraction buffer consisting of 4 M guanidine thiocyanate, 50 mM sodium citrate dihydrate, and 1 M Tris base as previously described^72^. Quality of RNA and DNA was assessed using a microvolume spectrophotometer (DS-11 Spectrophotometer; DeNovix) and only samples determined to have A260/280 absorbance ratios between 1.9 and 2.2 were considered for further analyses.

### Measurement of host gene expression and molecular quantification of bacterial communities

To determine host gene expression, a total of 1500 ng of the extracted RNA from adult head and gut samples was reverse transcribed to cDNA using a High-Capacity cDNA Reverse Transcription Kit following manufacturer’s instructions (Applied Biosystems, catalog number: 4368813). RT-qPCR reactions were performed with tenfold-diluted cDNA using the Power SYBR Green kit (Applied Biosystems) following manufacturer’s instructions. Oligonucleotide primers used to evaluate host expression of innate immune and antioxidant-related genes are listed in Supplementary Table 2. Honey bee *alpha-tubulin* was determined to be the most stably expressed endogenous control gene (compared *to ribosomal protein S5*, *microsomal glutathione-S-transferase*, and *UDP-glucuronyltransferase*) under experimental conditions in this study, and thus was chosen as the internal standard for normalization as per MIQE guidelines^73^. To measure bacterial abundance in samples, qPCR reactions were performed with tenfold-diluted DNA (from back extraction) using the Power SYBR Green kit (Applied Biosystems) following manufacturer’s instructions. The universal 16S rRNA gene, phylotype-specific, and species-specific primer sets that were used for molecule quantification of bacteria are listed in Supplementary Table 1. All qPCR reactions were performed in DNase- and RNase-free 384-well microplates using a QuantStudio 5 Real-Time PCR System (Applied Biosystems) and analyzed with associated QuantStudio Design and Analysis software. Relative gene expression was calculated using the 2^−ΔΔ^ Ct method^74^ whereas bacterial abundance (measured via copy number of target 16S rRNA genes) was calculated using previously established primer efficiencies and limits of detection^44,52,53^.

### Statistics and reproducibility

All compositional analyses on 16S rRNA gene sequencing datasets were performed using QIIME2 (v2020.2) or in R (v3.6.0) using ALDEx2 software (v1.18.0). Statistics for all other datasets were performed using GraphPad Prism (v8.3.0). Datasets with unique values were tested for normality using the Shapiro–Wilk test, whereas datasets with ties (two or more identical values) were tested for normality using the D’Agostino–Pearson test. Normally distributed data were statistically compared with two-tailed *t* tests, Pearson correlations, one-way ANOVAs, or two-way ANOVAs as indicated. ANOVA tests were complemented with Sidak’s multiple comparisons when appropriate. Nonparametric datasets were statistically compared using Wilcoxon, Mann–Whitney, or Kruskal–Wallis tests and complemented with Dunn’s multiple comparisons (quantitative datasets) or BH false discovery rate method (compositional datasets) when appropriate. Sample sizes and replicate details are described in the relevant ‘Methods’ sections.

### Reporting summary

Further information on research design is available in the Nature Research Reporting Summary linked to this article.

## Supplementary information

Supplementary Information

Description of Additional Supplementary Files

Supplementary Data 1

Supplementary Data 2

Supplementary Data 3

Reporting Summary

## Data Availability

Raw 16S rRNA gene sequencing reads were uploaded to the NCBI Sequence Read Archive and are available under BioProject accession: PRJNA610196. Other source data underlying applicable figures are available in Supplementary Datasets 1–3.
