# Supplementary material for: Lactobacillus spp. attenuate antibiotic-induced immune and microbiota dysregulation in honey bees
Source: Commun Biol. 2020 Sep 25;3:534. doi: 10.1038/s42003-020-01259-8 (PMC7519052; doi:10.1038/s42003-020-01259-8)
Supplement: Supplementary file 1 — Supplementary Information [file 42003_2020_1259_MOESM1_ESM.pdf]

Supplementary Information

Lactobacillus spp. attenuate antibiotic-induced immune and microbiota dysregulation in honey bees

Brendan A. Daisley[1,2], Andrew P. Pitek[3], John A. Chmiel[1,2], Shaeley Gibbons[1], Anna M. Chernyshova[3], Kait F. Al[1,2], Kyrillos M. Faragalla[3], Jeremy P. Burton[1,2,4], Graham J. Thompson[3], and Gregor Reid[1,2,4,#].

[1]Centre for Human Microbiome and Probiotic Research, Lawson Health Research Institute, London, Ontario, Canada; [2]Department of Microbiology and Immunology, The University of Western Ontario, London, Ontario, Canada; [3]Department of Biology, The University of Western Ontario, London, Ontario, Canada; [4]Department of Surgery, The University of Western Ontario, London, Ontario, Canada.

# Address correspondence to Dr. Gregor Reid, gregor@uwo.ca

This PDF file includes:

Supplementary Figures 1-4
Supplementary Tables 1-2

Other Supplementary Materials for this manuscript include the following:

Supplementary Datasets 1-3

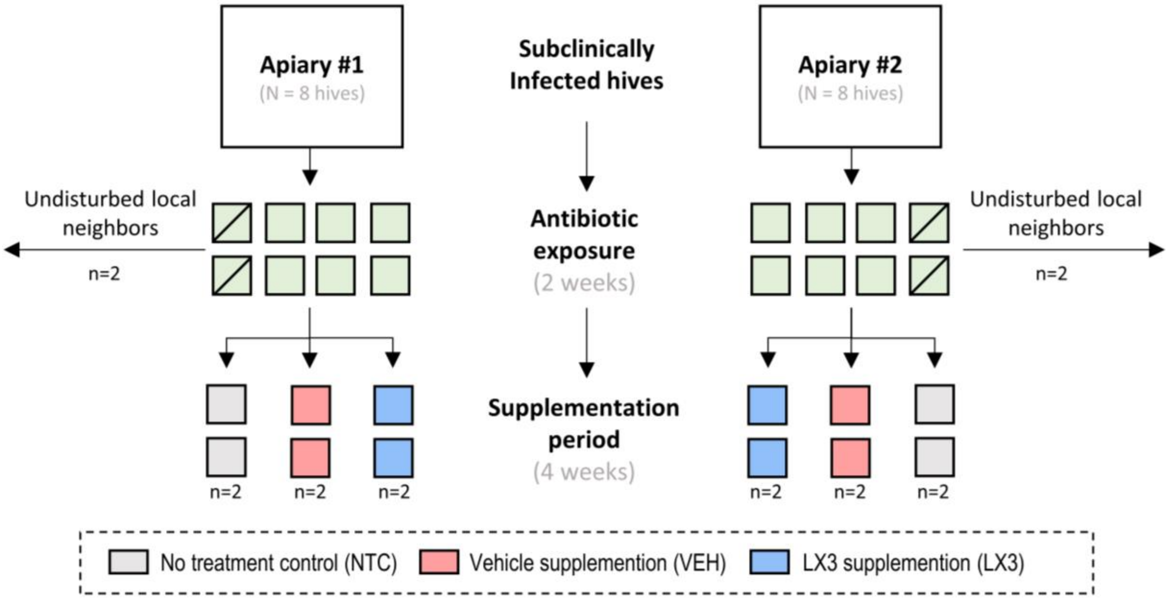

Supplementary Figure 1. Schematic diagram of experimental overview. Experimental apiaries were chosen on the basis of their inclusion within a boundary assessed to have a recent increase of AFB incidence as denoted through provincial apiary inspection by the Ontario Ministry of Agriculture and Food and Ministry of Rural Affairs. All hives received standard treatment with oxytetracycline hydrochloride (OTC) for two-weeks. Two hives in each apiary were then left to freely interact with experimental hives in an effort to emulate realistic buffering conditions of undisturbed local neighboring colonies. The remaining hives were longitudinally monitored for an additional four weeks while receiving either no treatment as a negative control (NTC), pollen patty supplementation as a vehicle control(VEH), or pollen patty with LX3 supplementation (LX3).

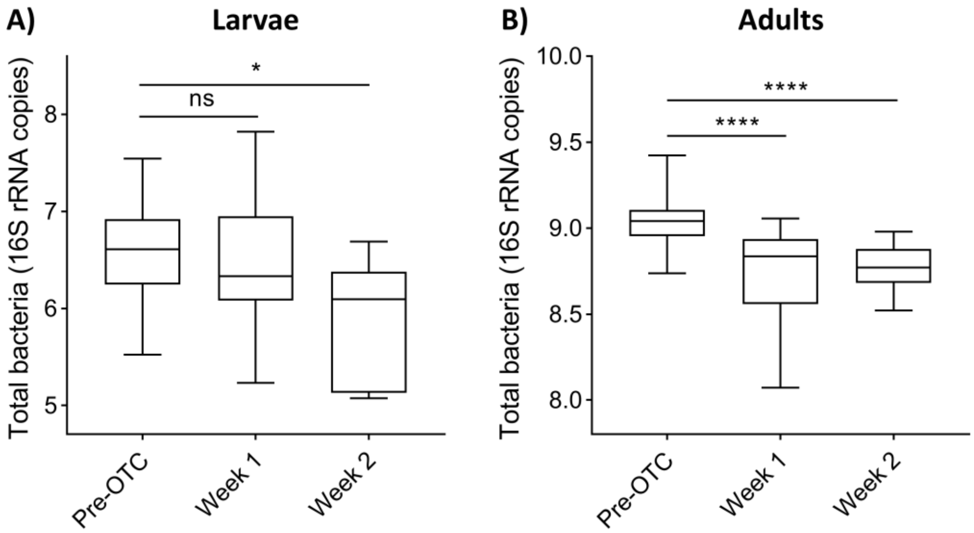

Supplementary Figure 2. Total bacterial loads in honey be adults and larvae during antibiotic treatment. Experimental hives were subjected to standard antibiotic treatment (OTC) for two weeks. Total bacterial loads in surface-sterilized **(A)** adults (dissected abdomens) and **(B)** larvae (whole bodies) were quantified via qPCR directly prior to hive administration with oxytetracycline (pre-abx) and a one-week intervals thereafter. Data represents the median (line in box), IQR (box), and minimum/maximum (whiskers) of n = 18 individual adult samples and n = 12 pooled larval samples (three larvae per pooled sampled) at each time point (Kruskal-Wallis with Dunn's multiple comparisons). ns = not significant, *P < 0.05, ****P < 0.0001.

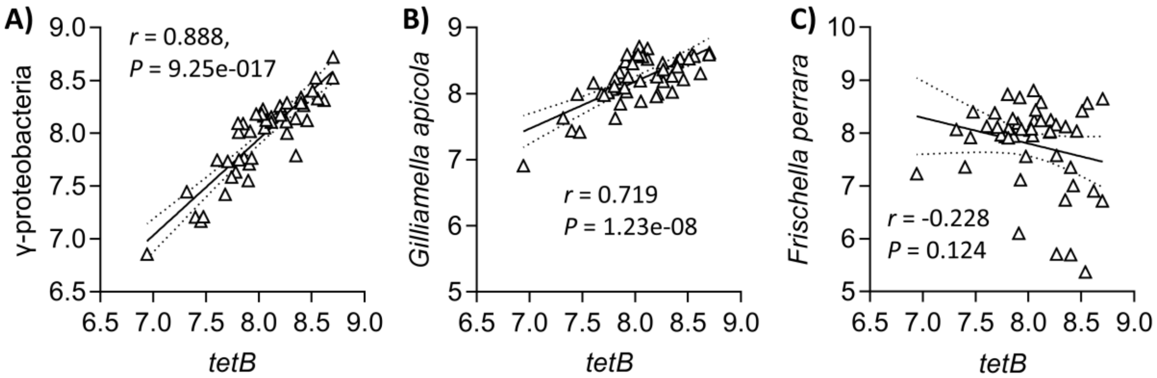

Supplementary Figure 3. Association between Gammaproteobacteria and *tetB* abundance in adult honey bees. After the six-week experimental period of antibiotic treatment followed by probiotic supplementation, intra-individual abundance of (A) total Gammaproteobacteria, (B) *Gilliamella apicola*, and (C) *Frischella perrara* was compared with *tetB* abundance. Data shown on log10 scale. *r* = Pearson correlation coefficient. N=48 adult gut samples. Simple linear regression (solid line) with 95% confidence interval (dotted lines) is shown for each.

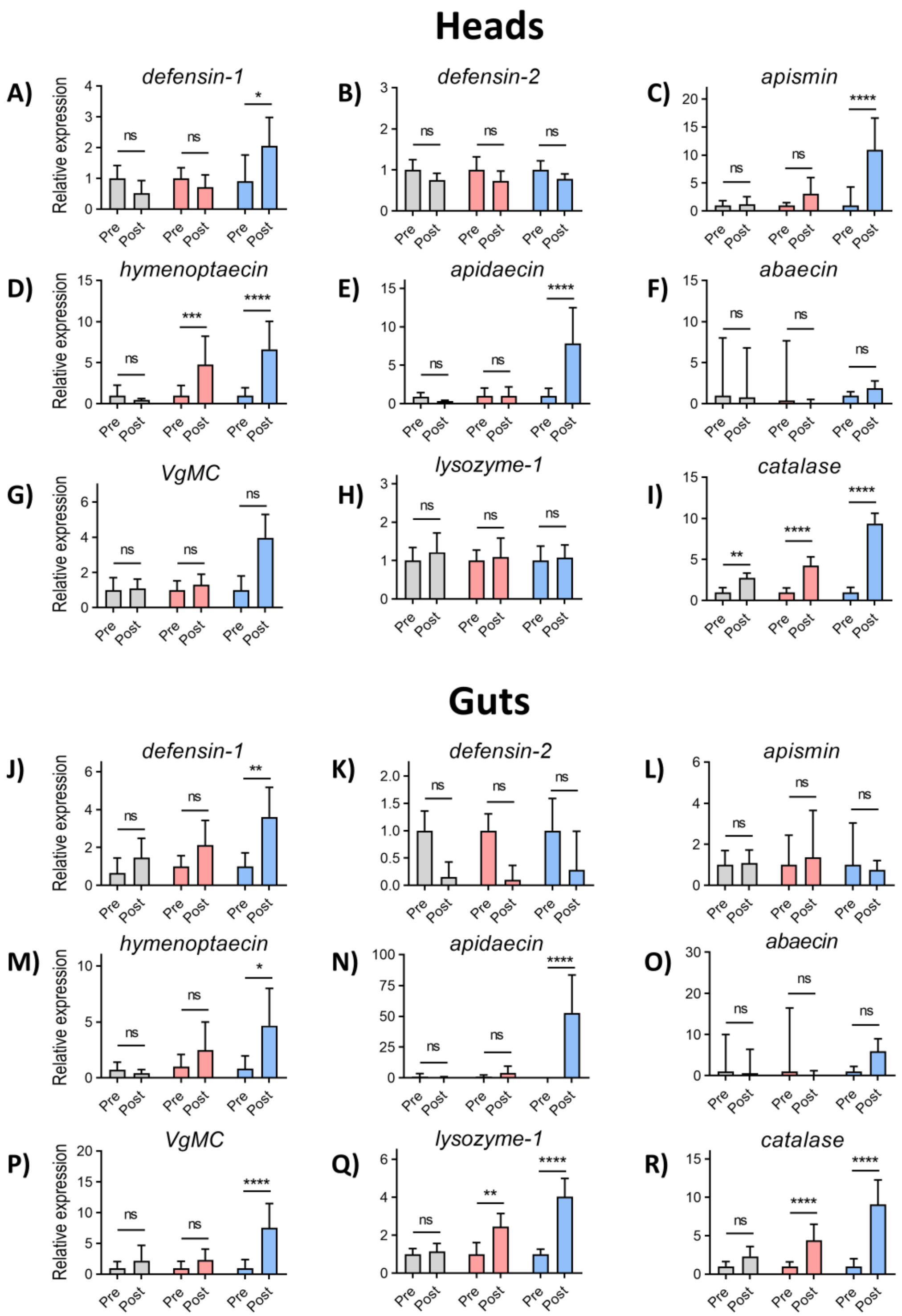

Supplementary Figure 4. Immune- and antioxidant-related gene expression in honey bee adults following in-hive LX3 supplementation. Hives received standard treatment with oxytetracycline hydrochloride for two weeks and then were longitudinally monitored for an additional four-week supplementation period in which hives received either no further treatment (NTC), pollen patty with vehicle supplementation (VEH), or pollen patty with LX3 supplementation (LX3). Expression of immune or antioxidant genes in the heads **(A-I)** and guts **(J-R)** of adult honey bees collected pre- and post-supplementation period were quantified via RT-qPCR. Mean ± standard deviation (two-way ANOVA with Sidak's multiple comparisons) of n=18 adult heads and n=18 adult guts per treatment group with technical duplicate repeats are shown. *P<0.05, **P<0.01, ***P<0.001, ****P<0.0001, ns = not significant.

Supplementary Tables

Supplementary Table 1. Primers used for qPCR-based quantification of bacteria.

Target	Forward Sequence (5'-3')	Reverse Sequence (5'-3')
B-actin	TTGTATGCCAACACTGTCCTTT	TGGCGCGATGATCTTAATTT
Rp5S	AATTATTTGGTCGCTGGAATTG	TAACGTCCAGCAGAATGTGGTA
Total bacteria	ACTCCTACGGGAGGCAGCAGT	ATTACCGCGGCTGCTGGC
Alphaproteobacteria	CIAGTGTAGAGGTGAAATTC	CCCCGTCAATTCCTTTGAGTT
Betaproteobacteria	CTTAGAGATAGGAGAGTG	TAATGATGGCAACTAATGACAA
Gammaproteobacteria	TCGTCAGCTCGTGTYGTGA	CGTAAGGGCCATGATG
Bifidobacterium	TACGGCCGCAAGGCTA	TCRTCCCCACCTTCCTCCG
Bacteroidetes	CRAACAGGATTAGATACCCT	GGTAAGGTTCCTCGCGTAT
Firmicutes	TGAAACTYAAAGGAATTGACG	ACCATGCACCACCTGTC
Paenibacillus larvae	CGGGAGACGCCAGGTTAG	TTCTTCCTTGGCAACAGAGC
Lactobacillus plantarum	ATTCATAGTCTAGTTGGAGGT	CCTGAACTGAGAGAATTTGA
Lactobacillus rhamnosus	TGCTTGCATCTTGATTTAATTTTG	GGTTCTTGGATYTATGCGGTATTAG
Lactobacillus kunkeei	GAGAAGCATTTACTAAGCCAAC	CATATTGACCTTTACCACCAGAT

Supplementary Table 2. Primers used for RT-qPCR determination of immune- and antioxidant-related gene expression.

**Target**	**Forward Sequence (5'-3')**	**Reverse Sequence (5'-3')**
Alpha-tubulin	GCACGTGAAGATCTAGCAGCTC	GCACCTTCTCCTTCACCTTCAG
Rp5S	AATTATTTGGTCGCTGGAATTG	TAACGTCCAGCAGAATGTGGTA
Microsomal glutathione-S-transferase	TTGCTCTGTAAGGTTGTTTTGC	TGTCTGGTTAACTACAAATCCTTCTG
Glucuronyltransferase	CACGGATACATCCTGCAGTCATC	GAGAATGACGAGATACAGAACTGTCAC
Defensin-1	TGCGCTGCTAACTGTCTCAG	AATGGCACTTAACCGAAACG
Defensin-2	GCAACTACCGCCTTTACGTC	GGGTAACGTGCGACGTTTTA
Hymenoptacein	CTCTTCTGTGCCGTTGCATA	CGTCTCCTGTCATTCCATT
Apisimin	TGAGCAAAATCGTTGCTGTC	AACGACATCCACGTTCGATT
VgMC	AGTTCCGACCGACGACGA	TTCCCTCCCACGGAGTCC
Apidaecin	TAGTCGCGGTATTTGGGAAT	TTTCACGTGCTTCATATTCTTCA
Abaecin	CAGCATTCGCATACGTACCA	GACCAGGAAACGTTGGAAAC
Catalase	GTCTTGGCCCAAACAATCTG	CATTCTCTAGGCCCACCAAA
Lysozyme	ACACGGTTGGTCACTGGTCC	GTCCCACGCTTTGAATCCCT
