# Supplementary material for: Lactobacillus spp. attenuate antibiotic-induced immune and microbiota dysregulation in honey bees
Source: Commun Biol. 2020 Sep 25;3:534. doi: 10.1038/s42003-020-01259-8 (PMC7519052; doi:10.1038/s42003-020-01259-8)
Supplement: Supplementary file 2 — Description of Additional Supplementary Files [file 42003_2020_1259_MOESM2_ESM.pdf]

Description of Additional Supplementary Files

File Name: Supplementary Data 1
Description: Source data for taxonomy of corresponding SVs for ALDEx2 effect plot of the adult honey bee microbiota during LX3 supplementation.

File Name: Supplementary Data 2
Description: Source data for immune- and antioxidant-related relative gene expression.

File Name: Supplementary Data 3
Description: Source data for intra-individual correlations between bacterial abundance and innate immune-related gene expression.
