# Supplementary material for: Lactobacillus spp. attenuate antibiotic-induced immune and microbiota dysregulation in honey bees
Source: Commun Biol. 2020 Sep 25;3:534. doi: 10.1038/s42003-020-01259-8 (PMC7519052; doi:10.1038/s42003-020-01259-8)
Supplement: Supplementary file 6 — Reporting Summary [file 42003_2020_1259_MOESM6_ESM.pdf]

nature research

Corresponding author(s): Gregor Reid
Last updated by author(s): Aug 3, 2020

Reporting Summary

Nature Research wishes to improve the reproducibility of the work that we publish. This form provides structure for consistency and transparency in reporting. For further information on Nature Research policies, see Authors & Referees and the Editorial Policy Checklist .

Statistics

For all statistical analyses, confirm that the following items are present in the figure legend, table legend, main text, or Methods section.

n/a	Confirmed	
☐	☒	The exact sample size (*n*) for each experimental group/condition, given as a discrete number and unit of measurement
☐	☒	A statement on whether measurements were taken from distinct samples or whether the same sample was measured repeatedly
☐	☒	The statistical test(s) used AND whether they are one- or two-sided<br>*Only common tests should be described solely by name; describe more complex techniques in the Methods section.*
☐	☒	A description of all covariates tested
☐	☒	A description of any assumptions or corrections, such as tests of normality and adjustment for multiple comparisons
☐	☒	A full description of the statistical parameters including central tendency (e.g. means) or other basic estimates (e.g. regression coefficient) AND variation (e.g. standard deviation) or associated estimates of uncertainty (e.g. confidence intervals)
☐	☒	For null hypothesis testing, the test statistic (e.g. *F*, *t*, *r*) with confidence intervals, effect sizes, degrees of freedom and *P* value noted<br>*Give P values as exact values whenever suitable.*
☐	☒	For Bayesian analysis, information on the choice of priors and Markov chain Monte Carlo settings
☐	☒	For hierarchical and complex designs, identification of the appropriate level for tests and full reporting of outcomes
☐	☒	Estimates of effect sizes (e.g. Cohen's *d*, Pearson's *r*), indicating how they were calculated

Our web collection on statistics for biologists contains articles on many of the points above.

Software and code

Policy information about availability of computer code

Data collection	Quant Studio 5 Real-Time PCR System (Applied Biosystems) software was used to collect qPCR data. MiSeq (Illumina) software was used to collect 16S rRNA sequencing data.
Data analysis	GraphPad Prism (v8.3) was used for statistical analyses throughout the manuscript with specific tests indicated in the methods section and figure legends . QuantStudio Design and Analysis software (Applied Biosytems) was used to analyze qPCR data. DADA2 pipeline with ALDEx2 and associated R software was used to to analyze 16S rRNA sequencing data (references to peer reviewed literature on these softwares are provided in the methods section).

For manuscripts utilizing custom algorithms or software that are central to the research but not yet described in published literature, software must be made available to editors/reviewers. We strongly encourage code deposition in a community repository (e.g. GitHub). See the Nature Research guidelines for submitting code & software for further information.

Data

Policy information about availability of data

All manuscripts must include a data availability statement. This statement should provide the following information, where applicable:

- Accession codes, unique identifiers, or web links for publicly available datasets
- A list of figures that have associated raw data
- A description of any restrictions on data availability

Raw sequence reads for 16S rRNA gene sequencing have been uploaded to the NCBI Sequence Read Archive and are accessible under BioProject ID PRJNA610196. Figure 2 and Figure 3 in the manuscript are associated with this raw data. There are no restrictions on data availability.

Field-specific reporting

Please select the one below that is the best fit for your research. If you are not sure, read the appropriate sections before making your selection.

☐ Life sciences ☐ Behavioural & social sciences ☒ Ecological, evolutionary & environmental sciences

For a reference copy of the document with all sections, see nature.com/documents/nr-reporting-summary-flat.pdf

Ecological, evolutionary & environmental sciences study design

All studies must disclose on these points even when the disclosure is negative.

Study description	Two separate experiments were performed in two distinct apiaries maintained within a single geographic region near Western University (London, Ontario, Canada). These experimental apiaries were selected based on their geographic inclusion within a boundary assess to have a recent increase of AFB incidence denoted through provincial apiary inspection by the Ontario Ministry of Agriculture and Food and Ministry of Rural Affairs (OMAFRA). Apiary A (N=8 hives) and apiary B (N=8 hives) were subjected to similar experimental designs (Supplementary Figure 1). In each case, all hives received standard treatment with oxytetracycline hydrochloride (catalog number: 0223111, MEDIVET Pharmaceuticals) for two weeks according to manufacturer instructions. Subsequently, hives were longitudinally monitored for an additional four weeks after being randomly assorted into the following experimental groups: (i) a no treatment control group (NTC) that received no supplementation following administration of OTC, (ii) a vehicle control group (VEH) that received a standard 250 g pollen substitute patty (28.5g of soy flour, 74.1g of granulated sucrose, 15.4g of debittered brewer's yeast, 132.1g of a 2:1 [w/v] simple sucrose-based syrup solution) with the addition of 4mL phosphate-buffered saline (0.01M) once per week, or (iii) a probiotic supplementation group (LX3) that received a 250 g pollen substitute patty infused with Lp39, LGR-1, and LkBR-1 (each at a final concentration of 1×109 colony forming units [CFU]/g) once per week. Samples of larvae (third-to-fifth instar) and adult nurse bees (found in close association with brood) were collected from the hives on a weekly basis for the six-week experimental period in apiary A and the four-week supplemental period for apiary B. Accordingly, we focused our downstream analyses recapitulating the effects of oxytetracycline on the adult gut microbiota on apiary A, whereas the combined experimental dataset from apiaries A and B were considered for all other analyses. Hive tools were flame sterilized prior to use between each of the hives and sterile latex gloves were employed to prevent cross-contamination of LX3 strains and potential pathogens.
Research sample	Honey bees (Apis mellifera) were the host insect species studied, though the main focus was on how associated bacterial species existing in their intestinal tract or exogenously supplemented could affect host health parameters. For adults bees, we evaluated only female "nurse" caste members as they are closely associated with brood area and provide a good representation of microbial diversity in the hive. Third-to-fifth instar larvae were chosen for evaluation on the basis that they harbor pathogens of interest in relation to the studied antibiotic, oxytetracycline.
Sampling strategy	Treatment groups were randomized in relation to physical orientation of hives in the apiary to buffer against environmental factors like edge effects and "robbing" behavior of honey bees. In addition, sampling occurred from four independent hives per treatment group across two temporally segregated sampling periods. This sampling procedure was chosen to ensure reproducibility of our findings at the hive level while accounting for the potential effects of any fluctuating environmental influence.
Data collection	AP, AC, and KF collected flash frozen honey bee samples in the field and transferred them to a - 80° freezer. BD, AP, JC, SG, and KA subsequently processed and analyzed the samples as described in the methods section for each of the experiments.
Timing and spatial scale	Experiments were performed during June-July 2018 for the first field trial and August-September 2018 for the second field trial.
Data exclusions	Standard quality assurance measures were performed on the 16S rRNA gene sequencing dataset in the DADA2 which resulted in removal of poor quality reads and chimeras. We have disclosed this in the methods sections and ensured that the raw sequencing reads that were uploaded to the NCBI Sequence Read Archive include these exclusions.
Reproducibility	Two field trials were performed with both demonstrating similar findings. In addition, we performed extensive sampling of individual honey bees from separate hives subjected to the same treatment thereby demonstrating reproducibility at the hive level as well. Moreover, because innate immune pathways are well conserved in insects, our findings in relation to the effect of bacteria on the honey bee immune system are expected to be highly reproducible in the context of different geographic localities albeit potential variation in environmental influencers.
Randomization	Treatment groups were randomly assigned to hives and sampling of individuals occurred via haphazardly collecting individuals located with the broad chamber of the hive.
Blinding	Samples were collected in containers labeled with alphanumerically codes corresponding to the hive they were collected from and then processed in the lab in random order. During subsequent data analysis steps, these labels were matched with the treatment received in order to perform appropriate statical tests between groups of interest.

Did the study involve field work? ☒ Yes ☐ No

Field work, collection and transport

Field conditions	Hive tools were flame sterilized prior to use between each of the hives and sterile latex gloves were employed to prevent cross-contamination of beneficial bacteria as well as potential pathogens. Samples were flash frozen and kept at - 80° until

downstream processing.

Location	This experiment was performed in London, Ontario Canada. Latitude: 43.02 N, Longitude: 81.28 W, Elevation: 251 m
Access and import/export	Not applicable.
Disturbance	There were no disturbances recorded during our study.

Reporting for specific materials, systems and methods

We require information from authors about some types of materials, experimental systems and methods used in many studies. Here, indicate whether each material, system or method listed is relevant to your study. If you are not sure if a list item applies to your research, read the appropriate section before selecting a response.

Materials & experimental systems

n/a	Involved in the study
☒	☐ Antibodies
☒	☐ Eukaryotic cell lines
☒	☐ Palaeontology
☐	☐ Animals and other organisms
☒	☐ Human research participants
☒	☐ Clinical data

Methods

n/a	Involved in the study
☒	☐ ChIP-seq
☒	☐ Flow cytometry
☒	☐ MRI-based neuroimaging

Animals and other organisms

Policy information about studies involving animals; ARRIVE guidelines recommended for reporting animal research

Laboratory animals	No laboratory animals were used.
Wild animals	Managed honey bees (Apis mellifera) were sampled from a total of N=16 hives via collection using sterile forceps followed by flash freezing in microtubes.
Field-collected samples	Sampling was performed on honey bees housed in standard Langstroth hives that were elevated ~36 inches above ground level using wooden support beams and were exposed to the natural weather patterns to emulate standard conditions in an apiary.
Ethics oversight	Ethics were not required for this study.

Note that full information on the approval of the study protocol must also be provided in the manuscript.
